# The genome sequence of the Blue-bordered Carpet moth *Plemyria rubiginata *(Denis & Schiffermüller) 1775

**DOI:** 10.12688/wellcomeopenres.21665.1

**Published:** 2024-05-16

**Authors:** Douglas Boyes, David C. Lees, Denise C. Wawman, University of Oxford and Wytham Woods Genome Acquisition Lab, Natural History Museum Genome Acquisition Lab, Darwin Tree of Life Barcoding collective, Wellcome Sanger Institute Tree of Life Management, Samples and Laboratory team, Wellcome Sanger Institute Scientific Operations: Sequencing Operations, Wellcome Sanger Institute Tree of Life Core Informatics team, Tree of Life Core Informatics collective, Darwin Tree of Life Consortium

**Affiliations:** 1 UK Centre for Ecology & Hydrology, Wallingford, England, UK; 2 Natural History Museum, London, England, UK; 3 Edward Grey Institute, Department of Biology, University of Oxford, Oxford, England, UK

**Keywords:** Plemyria rubiginata, Blue-bordered Carpet moth, genome sequence, chromosomal, Lepidoptera

## Abstract

We present a genome assembly from an individual female *Plemyria rubiginata* (the Blue-bordered Carpet moth; Arthropoda; Insecta; Lepidoptera; Geometridae). The genome sequence is 356.2 megabases in span. Most of the assembly is scaffolded into 30 chromosomal pseudomolecules, including the Z and W sex chromosomes. The mitochondrial genome has also been assembled and is 17.64 kilobases in length.

## Species taxonomy

Eukaryota; Opisthokonta; Metazoa; Eumetazoa; Bilateria; Protostomia; Ecdysozoa; Panarthropoda; Arthropoda; Mandibulata; Pancrustacea; Hexapoda; Insecta; Dicondylia; Pterygota; Neoptera; Endopterygota; Amphiesmenoptera; Lepidoptera; Glossata; Neolepidoptera; Heteroneura; Ditrysia; Obtectomera; Geometroidea; Geometridae; Larentiinae; *Plemyria*; *Plemyria rubiginata* (Denis & Schiffermüller) 1775 (NCBI:txid934957).

## Background

The Blue-bordered Carpet *Plemyria rubiginata* ([Denis & Schiffermüller], 1775) is a moth in the family Geometridae with a narrow forewing 12–15 mm long (typically 21–26 mm wingspan; Hausmann & Viidalepp, 2012).

Two subspecies have traditionally been recognised in the United Kingdom: *P. rubiginata rubiginata* with a whitish background and blue border, and a large brown blotch on the leading edge of the wing; and 'subspecies’ *P. rubiginata plumbata* (Curtis, 1837), distinguished by the brown blotch extending to become a full crossband, a darker and brownish border, and a ground colour of greyish-beige ( Waring *et al.*, 2017). However, the latter is internationally treated as f. *plumbata* (possibly an example of increasing northwards melanism at higher latitudes and elevations in northern England and Scotland, also in Norway and possibly Japan). By contrast, *P. rubiginata japonica* Inoue, 1955 is treated as validly distinct from the nominotypical subspecies from eastern Siberia and Mongolia to Japan ( Hausmann & Viidalepp, 2012). Randle *et al.* (2019) show a continuous distribution, although *P. rubiginata* is more scattered in Scotland and more localised in Ireland. Broadly, the species spans from 0–1600 m across almost the entire Palaearctic from north-western Portugal and Spain to China and Japan and the southern shores of the Mediterranean to northern Scandinavia and north-western Russia, with an isolated population in Turkey ( GBIF Secretariat, 2024; BOLD accessed 08/04/2024; Hausmann & Viidalepp, 2012).

Despite not being a species observed in large numbers, the Blue-bordered Carpet has shown a dramatic increase in abundance of 173% since 1970, although no change in distribution ( Randle *et al.*, 2019). The moth is univoltine, flying between early June and late September in the UK with some sign of a shift to an earlier modal abundance ( Randle *et al.*, 2019); and flying from mid-July at higher elevations in Europe ( Hausmann & Viidalepp, 2012).

*P. rubiginata* overwinters as an egg on the twigs of its foodplant, and the greenish larvae with yellow lateral stripes feed among spinnings on twig from April to June, preferring alder *Alnus glutinosa* (L.) Gaertn. or blackthorn *Prunus spinosa* L., but are also found on apple *Malus* spp., plum *Prunus* spp., hawthorn *Crataegus monogyna Jacq.* and birch *Betula* spp. ( Sterling *et al.*, 2012; Waring *et al.*, 2017) foodplants also utilised by the Nearctic sister species, *P. georgii* Hulst, 1896. ( Choi, 1998),

*P. rubiginata* occupies a single BIN cluster on BOLD (08/04/2024), BOLD:AAC1712, with up to 2.77% pairwise divergence within it ( *n* = 33), with Russian and Chinese exemplars showing highest divergences (~1.43% to the nearest exemplars). There are eight exemplars DNA barcoded from the UK (08/04/2024), the most northerly being from Lancashire but none so far from Scotland and Ireland, but at present there seems to be no mitochondrial evidence for genetic distinctiveness of northern UK populations. The Nearctic *P. georgii* (BOLD:AAB0097) by contrast is at least 5.65% divergent from it (although a species of *Chloroclystis*, BOLD:AFD5638, approaches *P. rubiginata* more closely at 4.17%).

The morphology and taxonomy of *Plemyria* Hübner, 1825 was reviewed by Choi (1998) and Hausmann & Viidalepp (2012), the genus comprising just the above two species. *Plemyria* belongs to the larentiinae tribe Cidariini. Based on a morphological analysis of adults and immatures, Choi (1997) placed the genus adjacent to *Chloroclystis* Hübner, 1825 and sister to *Thera* Stephens, 1831 + ( *Pennithera* Viidalepp, 1980 + *Heterothera* Inoue, 1943). The genus was not, however, included in the molecular larentiine phylogeny of Õunap *et al.* (2016).

The genome will be useful in investigating industrial melanism, stimulating more DNA barcoding, and clarifying the phylogeography of the species and the history of its sister species split, as well as establishing the phylogenetic placement of *Plemyria*.

## Genome sequence report

**Figure 1.**
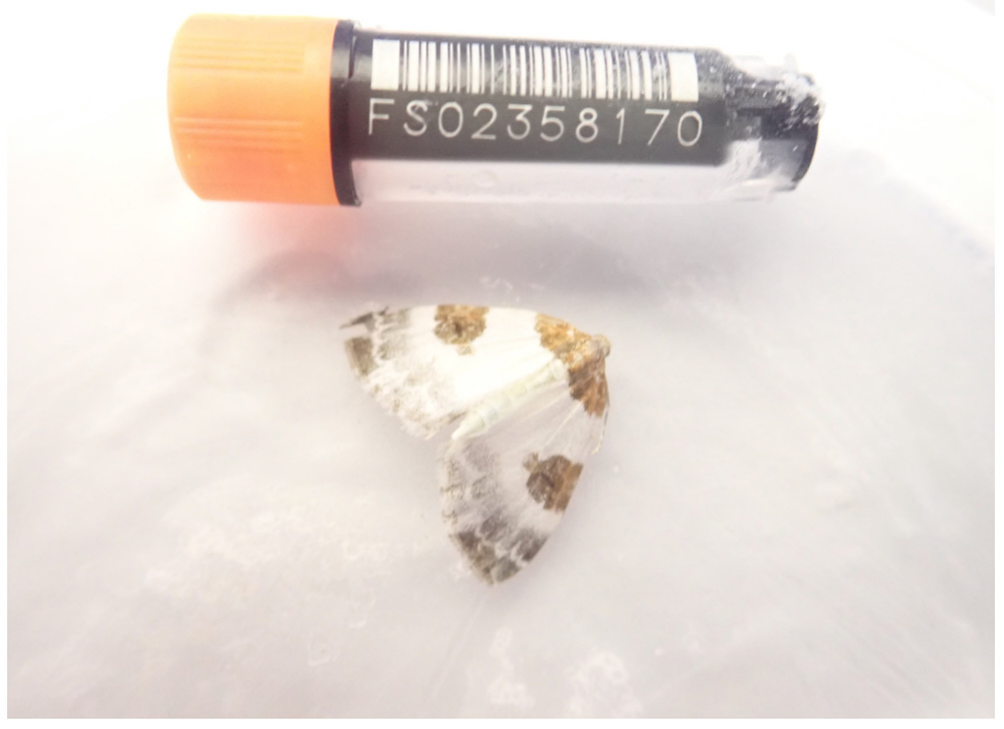
Photograph of the *Plemyria rubiginata* (ilPleRubi1) specimen used for genome sequencing.

The genome was sequenced from one female *Plemyria rubiginata* ( Figure 1) collected from Wytham Woods, Oxfordshire, UK (51.76, –1.34). A total of 94-fold coverage in Pacific Biosciences single-molecule HiFi long reads was generated. Primary assembly contigs were scaffolded with chromosome conformation Hi-C data. Manual assembly curation corrected 15 missing joins or mis-joins and removed 8 haplotypic duplications, reducing the assembly length by 1.07% and the scaffold number by 9.80%.

**Figure 2.**
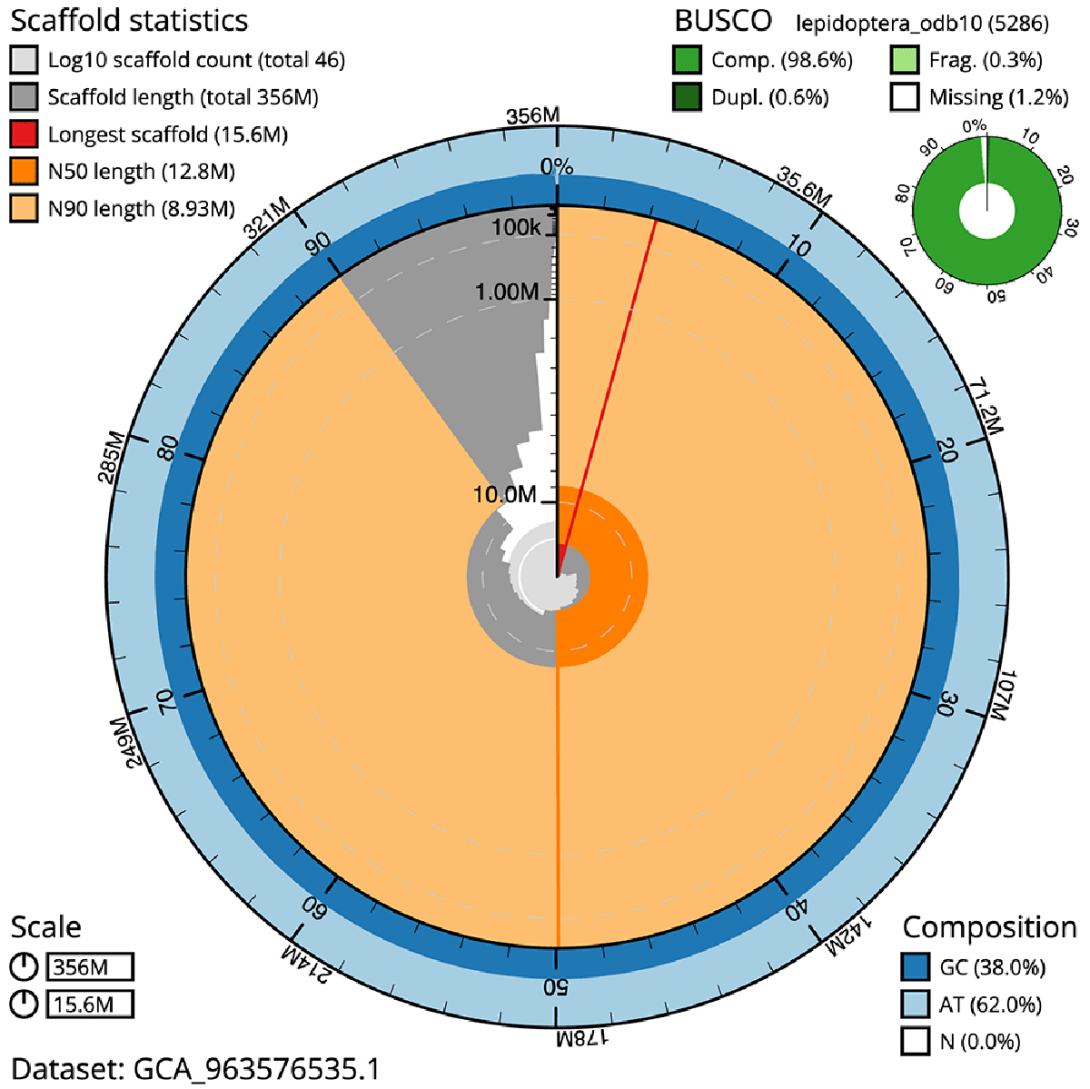
Genome assembly of *Plemyria rubiginata*, ilPleRubi1.1: metrics. The BlobToolKit snail plot shows N50 metrics and BUSCO gene completeness. The main plot is divided into 1,000 size-ordered bins around the circumference with each bin representing 0.1% of the 356,234,577 bp assembly. The distribution of scaffold lengths is shown in dark grey with the plot radius scaled to the longest scaffold present in the assembly (15,641,424 bp, shown in red). Orange and pale-orange arcs show the N50 and N90 scaffold lengths (12,846,343 and 8,926,186 bp), respectively. The pale grey spiral shows the cumulative scaffold count on a log scale with white scale lines showing successive orders of magnitude. The blue and pale-blue area around the outside of the plot shows the distribution of GC, AT and N percentages in the same bins as the inner plot. A summary of complete, fragmented, duplicated and missing BUSCO genes in the lepidoptera_odb10 set is shown in the top right. An interactive version of this figure is available at https://blobtoolkit.genomehubs.org/view/Plemyria_rubiginata/dataset/GCA_963576535.1/snail.

**Figure 3.**
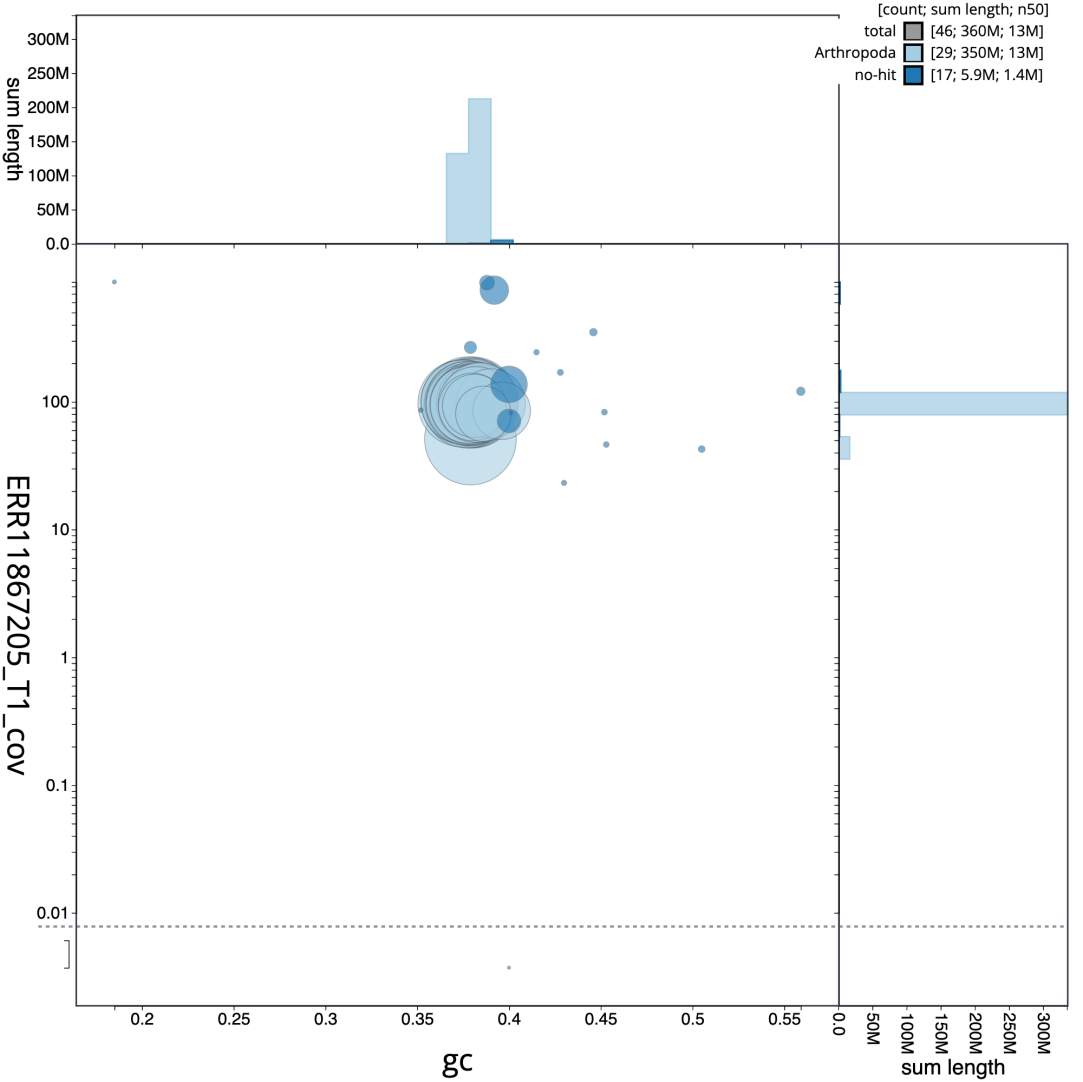
Genome assembly of *Plemyria rubiginata*, ilPleRubi1.1: BlobToolKit GC-coverage plot. Sequences are coloured by phylum. Circles are sized in proportion to sequence length. Histograms show the distribution of sequence length sum along each axis. An interactive version of this figure is available at https://blobtoolkit.genomehubs.org/view/Plemyria_rubiginata/dataset/GCA_963576535.1/blob.

**Figure 4.**
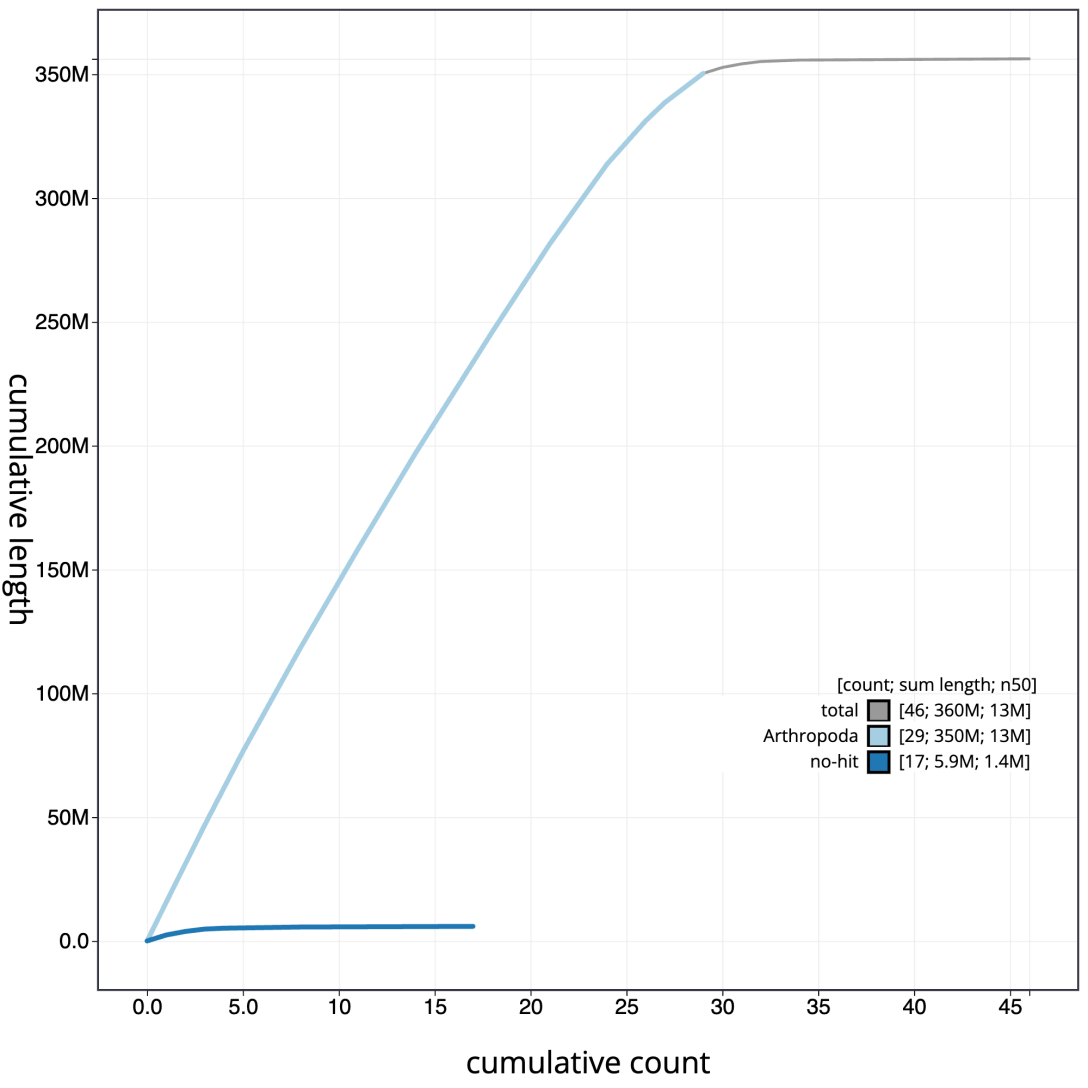
Genome assembly of *Plemyria rubiginata*, ilPleRubi1.1: BlobToolKit cumulative sequence plot. The grey line shows cumulative length for all sequences. Coloured lines show cumulative lengths of sequences assigned to each phylum using the buscogenes taxrule. An interactive version of this figure is available at https://blobtoolkit.genomehubs.org/view/Plemyria_rubiginata/dataset/GCA_963576535.1/cumulative.

**Figure 5.**
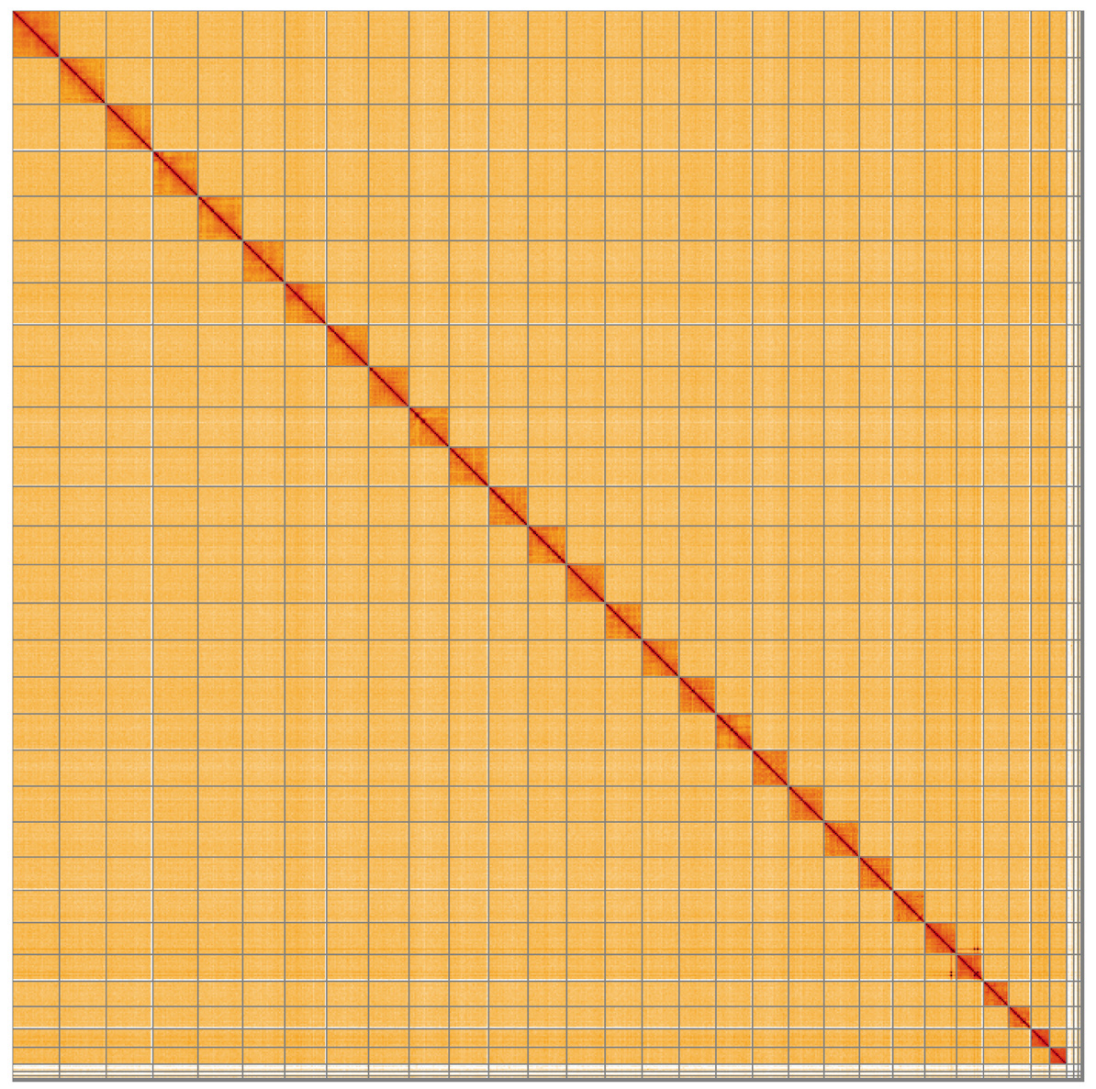
Genome assembly of *Plemyria rubiginata*, ilPleRubi1.1: Hi-C contact map of the ilPleRubi1.1 assembly, visualised using HiGlass. Chromosomes are shown in order of size from left to right and top to bottom. An interactive version of this figure may be viewed at https://genome-note-higlass.tol.sanger.ac.uk/l/?d=N6e0u10NSg-QOuCMZCLXhQ.

The final assembly has a total length of 356.2 Mb in 45 sequence scaffolds with a scaffold N50 of 12.8 Mb ( Table 1). The snail plot in Figure 2 provides a summary of the assembly statistics, while the distribution of assembly scaffolds on GC proportion and coverage is shown in Figure 3. The cumulative assembly plot in Figure 4 shows curves for subsets of scaffolds assigned to different phyla. Most (99.87%) of the assembly sequence was assigned to 30 chromosomal-level scaffolds, representing 28 autosomes and the Z and W sex chromosomes. Chromosome-scale scaffolds confirmed by the Hi-C data are named in order of size ( Figure 5; Table 2). While not fully phased, the assembly deposited is of one haplotype. Contigs corresponding to the second haplotype have also been deposited. The mitochondrial genome was also assembled and can be found as a contig within the multifasta file of the genome submission.

**Table 1.** Genome data for *Plemyria rubiginata*, ilPleRubi1.1.

Project accession data		
Assembly identifier	ilPleRubi1.1	
Species	*Plemyria rubiginata*	
Specimen	ilPleRubi1	
NCBI taxonomy ID	934957	
BioProject	PRJEB65207	
BioSample ID	SAMEA7701302	
Isolate information	ilPleRubi1, female: whole organism (DNA sequencing) ilPleRubi2: whole organism (Hi-C and RNA sequencing)	
Assembly metrics *		*Benchmark*
Consensus quality (QV)	63.1	*≥ 50*
*k*-mer completeness	100.0%	*≥ 95%*
BUSCO **	C:98.5%[S:97.9%,D:0.6%],F:0.3%,M:1.2%,n:5,286	*C ≥ 95%*
Percentage of assembly mapped to chromosomes	99.87%	*≥ 95%*
Sex chromosomes	ZW	*localised homologous pairs*
Organelles	Mitochondrial genome: 17.64 kb	*complete single alleles*
Raw data accessions		
PacificBiosciences Sequel IIe	ERR11867205, ERR11867206	
Hi-C Illumina	ERR11872574	
PolyA RNA-Seq Illumina	ERR12245586	
Genome assembly		
Assembly accession	GCA_963576535.1	
*Accession of alternate haplotype*	GCA_963576495.1	
Span (Mb)	356.2	
Number of contigs	68	
Contig N50 length (Mb)	11.8	
Number of scaffolds	45	
Scaffold N50 length (Mb)	12.8	
Longest scaffold (Mb)	15.64	

* Assembly metric benchmarks are adapted from column VGP-2020 of “Table 1: Proposed standards and metrics for defining genome assembly quality” from Rhie *et al.* (2021). ** BUSCO scores based on the lepidoptera_odb10 BUSCO set using version v5.4.3. C = complete [S = single copy, D = duplicated], F = fragmented, M = missing, n = number of orthologues in comparison. A full set of BUSCO scores is available at https://blobtoolkit.genomehubs.org/view/Plemyria_rubiginata/dataset/GCA_963576535.1/busco.

**Table 2.** Chromosomal pseudomolecules in the genome assembly of *Plemyria rubiginata*, ilPleRubi1.

INSDC accession	Chromosome	Length (Mb)	GC%
OY754906.1	1	15.54	38.0
OY754907.1	2	15.54	38.0
OY754908.1	3	14.99	38.0
OY754909.1	4	14.82	38.0
OY754910.1	5	13.99	37.5
OY754911.1	6	13.97	38.0
OY754912.1	7	13.87	37.5
OY754913.1	8	13.47	38.0
OY754914.1	9	13.31	37.5
OY754915.1	10	13.13	37.5
OY754916.1	11	13.09	37.5
OY754917.1	12	12.85	38.0
OY754918.1	13	12.76	38.0
OY754919.1	14	12.3	37.5
OY754920.1	15	12.3	37.5
OY754921.1	16	12.25	38.0
OY754922.1	17	12.12	38.5
OY754923.1	18	11.96	38.0
OY754924.1	19	11.83	38.0
OY754925.1	20	11.73	38.0
OY754926.1	21	11.09	38.0
OY754927.1	22	10.6	38.5
OY754928.1	23	10.65	38.0
OY754929.1	24	8.93	39.0
OY754930.1	25	8.44	38.0
OY754931.1	26	7.39	38.0
OY754932.1	27	6.13	39.5
OY754933.1	28	5.64	38.5
OY754934.1	W	2.41	40.0
OY754905.1	Z	15.64	38.0
OY754935.1	MT	0.02	18.5

The estimated Quality Value (QV) of the final assembly is 63.1 with *k*-mer completeness of 100.0%, and the assembly has a BUSCO v completeness of 98.5% (single = 97.9%, duplicated = 0.6%), using the lepidoptera_odb10 reference set ( *n* = 5,286).

Metadata for specimens, BOLD barcode results, spectra estimates, sequencing runs, contaminants and pre-curation assembly statistics are given at https://links.tol.sanger.ac.uk/species/934957.

## Methods

### Sample acquisition and nucleic acid extraction

A female *Plemyria rubiginata* (specimen ID Ox000535, ToLID ilPleRubi1) was collected from Wytham Woods, Oxfordshire (biological vice-county Berkshire), UK (latitude 51.76, longitude –1.34) on 2020-06-25 using a light trap. The specimen was collected and identified by Douglas Boyes (University of Oxford) and preserved on dry ice.

The specimen used for Hi-C and RNA sequencing (specimen ID NHMUK014584832, ToLID ilPleRubi2) was hand-picked in Lucas Wood, High Wycombe, London (latitude 51.63, longitude –0.74) on 2022-06-23. The specimen was collected and identified by David Lees (Natural History Museum) and preserved by dry-freezing at –80 °C.

The workflow for high molecular weight (HMW) DNA extraction at the Wellcome Sanger Institute (WSI) Tree of Life Core Laboratory includes a sequence of core procedures: sample preparation; sample homogenisation, DNA extraction, fragmentation, and clean-up. In sample preparation, the ilPleRubi1 sample was weighed and dissected on dry ice ( Jay *et al.*, 2023). Tissue from the whole organism was homogenised using a PowerMasher II tissue disruptor ( Denton *et al.*, 2023a).

HMW DNA was extracted using the Automated MagAttract v1 protocol ( Sheerin *et al.*, 2023). DNA was sheared into an average fragment size of 12–20 kb in a Megaruptor 3 system with speed setting 30 ( Todorovic *et al.*, 2023). Sheared DNA was purified by solid-phase reversible immobilisation ( Strickland *et al.*, 2023): in brief, the method employs a 1.8X ratio of AMPure PB beads to sample to eliminate shorter fragments and concentrate the DNA. The concentration of the sheared and purified DNA was assessed using a Nanodrop spectrophotometer and Qubit Fluorometer and Qubit dsDNA High Sensitivity Assay kit. Fragment size distribution was evaluated by running the sample on the FemtoPulse system.

RNA was extracted from tissue of ilPleRubi2 in the Tree of Life Laboratory at the WSI using the RNA Extraction: Automated MagMax™ *mir*Vana protocol ( do Amaral *et al.*, 2023). The RNA concentration was assessed using a Nanodrop spectrophotometer and a Qubit Fluorometer using the Qubit RNA Broad-Range Assay kit. Analysis of the integrity of the RNA was done using the Agilent RNA 6000 Pico Kit and Eukaryotic Total RNA assay.

Protocols developed by the WSI Tree of Life laboratory are publicly available on protocols.io ( Denton *et al.*, 2023b).

### Sequencing

Pacific Biosciences HiFi circular consensus DNA sequencing libraries were constructed according to the manufacturers’ instructions. Poly(A) RNA-Seq libraries were constructed using the NEB Ultra II RNA Library Prep kit. DNA and RNA sequencing was performed by the Scientific Operations core at the WSI on Pacific Biosciences Sequel IIe (HiFi) and Illumina NovaSeq 6000 (RNA-Seq) instruments. Hi-C data were also generated from tissue of ilPleRubi2 using the Arima2 kit and sequenced on the Illumina NovaSeq 6000 instrument.

### Genome assembly and curation

Assembly was carried out with Hifiasm ( Cheng *et al.*, 2021) and haplotypic duplication was identified and removed with purge_dups ( Guan *et al.*, 2020). The assembly was then scaffolded with Hi-C data ( Rao *et al.*, 2014) using YaHS ( Zhou *et al.*, 2023). The assembly was checked for contamination and corrected using the TreeVal pipeline ( Pointon *et al.*, 2023). Manual curation was performed using JBrowse2 ( Diesh *et al.*, 2023), HiGlass ( Kerpedjiev *et al.*, 2018) and PretextView ( Harry, 2022). The mitochondrial genome was assembled using MitoHiFi ( Uliano-Silva *et al.*, 2023), which runs MitoFinder ( Allio *et al.*, 2020) or MITOS ( Bernt *et al.*, 2013) and uses these annotations to select the final mitochondrial contig and to ensure the general quality of the sequence.

### Final assembly evaluation

The final assembly was post-processed and evaluated with the three Nextflow ( Di Tommaso *et al.*, 2017) DSL2 pipelines “sanger-tol/readmapping” ( Surana *et al.*, 2023a), “sanger-tol/genomenote” ( Surana *et al.*, 2023b), and “sanger-tol/blobtoolkit” ( Muffato *et al.*, 2024). The pipeline sanger-tol/readmapping aligns the Hi-C reads with bwa-mem2 ( Vasimuddin *et al.*, 2019) and combines the alignment files with SAMtools ( Danecek *et al.*, 2021). The sanger-tol/genomenote pipeline transforms the Hi-C alignments into a contact map with BEDTools ( Quinlan & Hall, 2010) and the Cooler tool suite ( Abdennur & Mirny, 2020), which is then visualised with HiGlass ( Kerpedjiev *et al.*, 2018). It also provides statistics about the assembly with the NCBI datasets ( Sayers *et al.*, 2024) report, computes *k*-mer completeness and QV consensus quality values with FastK and MerquryFK, and a completeness assessment with BUSCO ( Manni *et al.*, 2021).

The sanger-tol/blobtoolkit pipeline is a Nextflow port of the previous Snakemake Blobtoolkit pipeline ( Challis *et al.*, 2020). It aligns the PacBio reads with SAMtools and minimap2 ( Li, 2018) and generates coverage tracks for regions of fixed size. In parallel, it queries the GoaT database ( Challis *et al.*, 2023) to identify all matching BUSCO lineages to run BUSCO ( Manni *et al.*, 2021). For the three domain-level BUSCO lineage, the pipeline aligns the BUSCO genes to the Uniprot Reference Proteomes database ( Bateman *et al.*, 2023) with DIAMOND ( Buchfink *et al.*, 2021) blastp. The genome is also split into chunks according to the density of the BUSCO genes from the closest taxonomically lineage, and each chunk is aligned to the Uniprot Reference Proteomes database with DIAMOND blastx. Genome sequences that have no hit are then chunked with seqtk and aligned to the NT database with blastn ( Altschul *et al.*, 1990). All those outputs are combined with the blobtools suite into a blobdir for visualisation.

All three pipelines were developed using the nf-core tooling ( Ewels *et al.*, 2020), use MultiQC ( Ewels *et al.*, 2016), and make extensive use of the Conda package manager, the Bioconda initiative ( Grüning *et al.*, 2018), the Biocontainers infrastructure ( da Veiga Leprevost *et al.*, 2017), and the Docker ( Merkel, 2014) and Singularity ( Kurtzer *et al.*, 2017) containerisation solutions.

Table 3 contains a list of relevant software tool versions and sources.

**Table 3.** Software tools: versions and sources.

Software tool	Version	Source
BEDTools	2.30.0	https://github.com/arq5x/bedtools2
Blast	2.14.0	ftp://ftp.ncbi.nlm.nih.gov/blast/executables/blast+/
BlobToolKit	4.3.7	https://github.com/blobtoolkit/blobtoolkit
BUSCO	5.4.3 and 5.5.0	https://gitlab.com/ezlab/busco
bwa-mem2	2.2.1	https://github.com/bwa-mem2/bwa-mem2
Cooler	0.8.11	https://github.com/open2c/cooler
DIAMOND	2.1.8	https://github.com/bbuchfink/diamond
fasta_windows	0.2.4	https://github.com/tolkit/fasta_windows
FastK	427104ea91c78c3b8b8b49f1a7d6bbeaa869ba1c	https://github.com/thegenemyers/FASTK
GoaT CLI	0.2.5	https://github.com/genomehubs/goat-cli
Hifiasm	0.19.5-r587	https://github.com/chhylp123/hifiasm
HiGlass	44086069ee7d4d3f6f3f0012569789ec138f42b84aa44357826c0b6753eb28de	https://github.com/higlass/higlass
MerquryFK	d00d98157618f4e8d1a9190026b19b471055b22e	https://github.com/thegenemyers/MERQURY.FK
MitoHiFi	3	https://github.com/marcelauliano/MitoHiFi
MultiQC	1.14, 1.17, and 1.18	https://github.com/MultiQC/MultiQC
NCBI Datasets	15.12.0	https://github.com/ncbi/datasets
Nextflow	23.04.0-5857	https://github.com/nextflow-io/nextflow
PretextView	0.2	https://github.com/wtsi-hpag/PretextView
purge_dups	1.2.5	https://github.com/dfguan/purge_dups
samtools	1.16.1, 1.17, and 1.18	https://github.com/samtools/samtools
sanger-tol/genomenote	1.1.1	https://github.com/sanger-tol/genomenote
sanger-tol/readmapping	1.2.1	https://github.com/sanger-tol/readmapping
Seqtk	1.3	https://github.com/lh3/seqtk
Singularity	3.9.0	https://github.com/sylabs/singularity
TreeVal	1.0.0	https://github.com/sanger-tol/treeval
YaHS	1.2a.2	https://github.com/c-zhou/yahs

### Wellcome Sanger Institute – Legal and Governance

The materials that have contributed to this genome note have been supplied by a Darwin Tree of Life Partner. The submission of materials by a Darwin Tree of Life Partner is subject to the **‘Darwin Tree of Life Project Sampling Code of Practice’**, which can be found in full on the Darwin Tree of Life website here. By agreeing with and signing up to the Sampling Code of Practice, the Darwin Tree of Life Partner agrees they will meet the legal and ethical requirements and standards set out within this document in respect of all samples acquired for, and supplied to, the Darwin Tree of Life Project.

Further, the Wellcome Sanger Institute employs a process whereby due diligence is carried out proportionate to the nature of the materials themselves, and the circumstances under which they have been/are to be collected and provided for use. The purpose of this is to address and mitigate any potential legal and/or ethical implications of receipt and use of the materials as part of the research project, and to ensure that in doing so we align with best practice wherever possible. The overarching areas of consideration are:

•   Ethical review of provenance and sourcing of the material

•   Legality of collection, transfer and use (national and international)

Each transfer of samples is further undertaken according to a Research Collaboration Agreement or Material Transfer Agreement entered into by the Darwin Tree of Life Partner, Genome Research Limited (operating as the Wellcome Sanger Institute), and in some circumstances other Darwin Tree of Life collaborators.

## Data Availability

European Nucleotide Archive: *Plemyria rubiginata* (blue-bordered carpet). Accession number PRJEB65207; https://identifiers.org/ena.embl/PRJEB65207 ( Wellcome Sanger Institute, 2023). The genome sequence is released openly for reuse. The *Plemyria rubiginata* genome sequencing initiative is part of the Darwin Tree of Life (DToL) project. All raw sequence data and the assembly have been deposited in INSDC databases. The genome will be annotated using available RNA-Seq data and presented through the Ensembl pipeline at the European Bioinformatics Institute. Raw data and assembly accession identifiers are reported in Table 1.

## Author information

Members of the University of Oxford and Wytham Woods Genome Acquisition Lab are listed here: https://doi.org/10.5281/zenodo.7125292.

Members of the Natural History Museum Genome Acquisition Lab are listed here: https://doi.org/10.5281/zenodo.7139035.

Members of the Darwin Tree of Life Barcoding collective are listed here: https://doi.org/10.5281/zenodo.4893703.

Members of the Wellcome Sanger Institute Tree of Life Management, Samples and Laboratory team are listed here: https://doi.org/10.5281/zenodo.10066175.

Members of Wellcome Sanger Institute Scientific Operations: Sequencing Operations are listed here: https://doi.org/10.5281/zenodo.10043364.

Members of the Wellcome Sanger Institute Tree of Life Core Informatics team are listed here: https://doi.org/10.5281/zenodo.10066637.

Members of the Tree of Life Core Informatics collective are listed here: https://doi.org/10.5281/zenodo.5013541.

Members of the Darwin Tree of Life Consortium are listed here: https://doi.org/10.5281/zenodo.4783558.
