# Supplementary material for: The genome sequence of the Blue-bordered Carpet moth Plemyria rubiginata (Denis & Schiffermüller) 1775
Source: Wellcome Open Res. 2024 May 16;9:271. [Version 1] doi: 10.12688/wellcomeopenres.21665.1 (PMC11415762; doi:10.12688/wellcomeopenres.21665.1)
Supplement: Peer review history

# Reviewer report: Reviewer response for version 1

**DOI:** 10.21956/wellcomeopenres.23938.r99779

**Published:** 2024-09-20

**Authors:** Varun Arya

**Affiliations:** 1 Entomology & Agricultural Zoology, Banaras Hindu University (Ringgold ID: 30114), Varanasi, Uttar Pradesh, India

This study presents a comprehensive genomic assembly of *Plemyria rubiginata*, commonly referred to as the 'blue-bordered carpet moth.' As present in the manuscript, the assembled sequences were successfully mapped to both autosomes and sex chromosomes.

**Minor comments:**

- Please make the following changes in the keywords: italicize “ *Plemyria rubiginata*.” Keep “Blue-bordered Carpet moth” in lower case, also in other places in the manuscript while writing. The name of the insect orders, if used in the middle of the sentence, shall not be capitalized. Write “Lepidoptera” in lower case.
- In the background: instead of “([Denis & Schiffermüller], 1775)”, write it as “(Denis & Schiffermüller, 1775)”. Also correct it in the title. In the manuscript, “However, the latter is internationally treated as *f. plumbata*”, the identity of “ *f. plumbata*” is not clear if the authors have mentioned it as a distinct sub species or is it a typing error. The citation of “([Denis & Schiffermüller], 1775)” can be rewritten as “(Denis & Schiffermüller, 1775)” in both the background and the title.

Are sufficient details of methods and materials provided to allow replication by others?

Yes

Is the rationale for creating the dataset(s) clearly described?

Yes

Are the datasets clearly presented in a useable and accessible format?

Yes

Are the protocols appropriate and is the work technically sound?

Yes

Reviewer Expertise:

Entomology & Agricultural Zoology, Plant Protection, Molecular Entomology, Insect Ecology

I confirm that I have read this submission and believe that I have an appropriate level of expertise to confirm that it is of an acceptable scientific standard.

# Reviewer report: Reviewer response for version 1

**DOI:** 10.21956/wellcomeopenres.23938.r97168

**Published:** 2024-09-10

**Authors:** Nathan P Havill

**Affiliations:** 1 Northern Research Station, Hamden, USA

The article, "The genome sequence of the Blue-bordered Carpet moth *Plemyria rubiginata..." *reports a genome high quality assembly for a female individual of this species. The methods and analyses appear to be robust, and the genome sequence is a welcome addition to the growing number of lepidopteran examples. I have only minor edits to suggest below:

**Title:** Do not capitalize “Blue” and “Carpet”. Also, change to, “… *Plemyria rubiginata* (Denis & Schiffermüller, 1775).

**Keywords:** Please replace the words that are already in the title with others.

**Background:**

- Change to, “… *Plemyria rubiginata* (Denis & Schiffermüller, 1775).
- Instead of “.. treated as f. *plumbata,*” do you mean “treated as *P. plumbata*” ?
- This might be better: “By contrast, P *. rubiginata japonica* Inoue, 1955, from eastern Siberia and Mongolia to Japan, is treated as validly distinct from the nominotypical subspecies (Hausmann & Viidalepp, 2012).
- From the sentence construction, is not clear which food plants are also utilized by *P. georgii*. All of them? Please make this more clear.
- This might be better: “The morphology and taxonomy of *Plemyria* Hübner, 1825, the genus comprising just the above two species, was reviewed by Choi (1998) and Hausmann & Viidalepp (2012).”

Are sufficient details of methods and materials provided to allow replication by others?

Yes

Is the rationale for creating the dataset(s) clearly described?

Yes

Are the datasets clearly presented in a useable and accessible format?

Yes

Are the protocols appropriate and is the work technically sound?

Yes

Reviewer Expertise:

Insect population genetics and phylogenetics.

I confirm that I have read this submission and believe that I have an appropriate level of expertise to confirm that it is of an acceptable scientific standard.
